# Evaluating Canada’s initiative of enhanced screening for tuberculosis infection in migrants: Implementation lessons from Alberta

**DOI:** 10.14745/ccdr.v51i101112a02

**Published:** 2025-12-12

**Authors:** Courtney Heffernan, Abdul Jamro, Mary Lou Egedahl, Richard Long

**Affiliations:** 1 Tuberculosis Program Evaluation and Research Unit, Department of Medicine, Faculty of Medicine and Dentistry, University of Alberta, Edmonton, AB; 2 School of Public Health, University of Alberta, Edmonton, AB

**Keywords:** tuberculosis, surveillance, incidence rate, migrants, screening, evaluation, implementation

## Abstract

**Background:**

The domestic tuberculosis (TB) disease burden in high-income, low TB-incidence countries is largely driven by the reactivation of remotely acquired TB infections (TBIs) in people born outside the country (PBOC). In Canada, PBOC now accounts for more than three quarters of annual active TB diagnoses. To prevent some of this disease experience, Immigration, Refugees and Citizenship Canada (IRCC) rolled out a new TBI screening initiative in 2019.

**Objective:**

An evaluation of TB outcomes among individuals referred through this initiative between May 2019 and May 2023 in Alberta, Canada.

**Methods:**

Inclusion criteria for this initiative are migrants who are required to undergo an immigration medical exam with at least one of HIV/AIDS, solid organ transplant, end-stage renal disease, recent close TB contact (within five years), and past head and neck cancer. Those with a positive screening test for TBI are referred directly to TB services in the stated province/territory of landing for assessment and treatment.

**Results:**

Over four years, 179 referrals were made to Alberta. No one referred through the program and offered treatment developed active TB. Overall, 95 individuals were considered suitable candidates for prevention, among whom 87% accepted. Completion was high at nearly 95%. Inefficiencies included 113 individuals undergoing repeated TBI testing locally, 39 (21.8%) referrals not meeting the inclusion criteria, and 61 (34.1%) individuals being rereferred despite being past patients of Alberta TB services.

**Conclusion:**

Our findings highlight that, in Alberta, IRCC’s new TBI screening initiative was highly successful in connecting referred individuals to TB services. The initiative experienced some inefficiencies and we describe areas where it could be improved.

## Introduction

Despite being preventable and curable, in 2023, 10.8 million people fell ill with tuberculosis (TB) disease worldwide and 1.25 million succumbed to its effects ((1)). Tuberculosis has traditionally been conceived as existing in a binary of TB infection (TBI) and TB disease (TBD), with the former being neither contagious nor symptomatic but requisite to developing TBD ((2)). The global prevalence of TBI is estimated at 25%, but only 5%–10% of those infected will progress to disease ((3)). Progression risk is highest among people who have been recently infected, have immune compromising conditions, or are in poor general health, including from undernutrition ((4)). As a result, TBD proliferates in places where the health and social welfare needs of most citizens are largely unmet, as in low- and middle-income countries. Meanwhile, in high-income countries, the majority of TBD is experienced by people born outside the country (PBOC), with most resulting from reactivation of remotely acquired infections ((5)).

**Figure 1:**
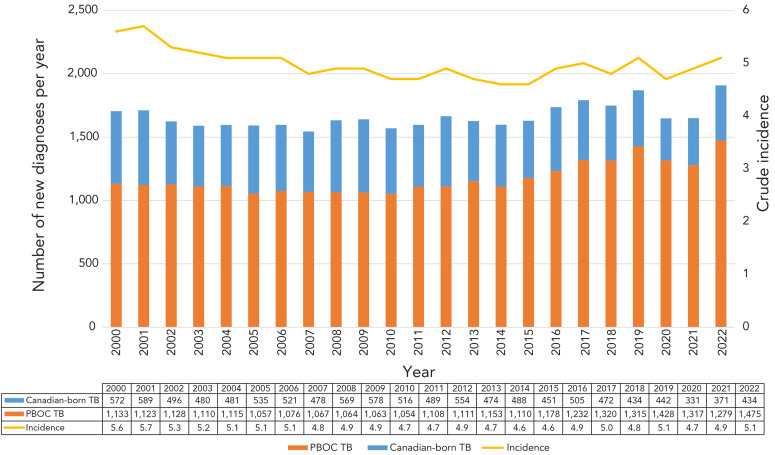
Annual count of individuals with tuberculosis disease who are Canadian-born and people born outside the country along with the overall tuberculosis incidence rate in Canada, 2000–2022 Abbreviations: PBOC, people born outside the country; TB, tuberculosis

Canada has a rate of TBD that is among the lowest in the world, but which has hovered at approximately five per 100,000 population from 2000 to present ((6)). In part, this may be because in 2016, the decades long shift from people arriving from countries of Western Europe with a low TB burden to people arriving from countries of Asia, Africa, and Latin America with intermediate and high TB incidence was at a nearly 30/70 split, while the absolute number of migrants have been increasing rapidly since ((7–10)). By 2019, PBOC made up 74% of all people affected by TB nationally, see **Figure 1**. Such considerations imply that the immigration pathways are ideal settings for TBI screening with positive impacts to both the individual and public.

**Figure 2:**
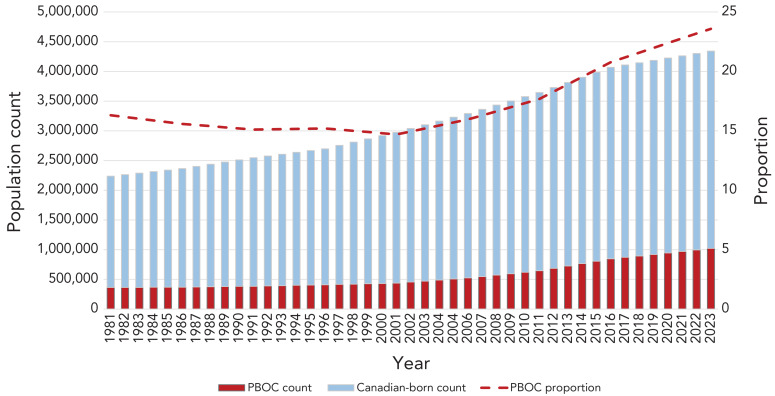
Alberta population count by group along with the proportion who are people born outside the country, 1981–2023 Abbreviation: PBOC, people born outside the country Source: Population estimates from Statistics Canada (https://www.statcan.gc.ca/)

Canada has high levels of immigration, having welcomed 437,000 new permanent residents in 2022 ((11)). In light of the ostensible TB prevention gap among PBOC to Canada, Immigration, Refugees and Citizenship Canada (IRCC) introduced a federal program of enhanced screening for TBI in 2019 as an add-on to their existing medical surveillance program for TBD. Prior to the introduction of this initiative, the immigration focus was on finding active TBD, including domestic post-landing medical surveillance for TB among those with abnormal chest X-rays or past history of TB ((12)). This article describes TB outcomes among individuals referred by this new, systematic TBI screening initiative between May 2019 and May 2023 to the province of Alberta, where net international migration is a major component of population growth and the majority of TBD is diagnosed among PBOC ((13,14)). The top three countries of birth of newcomers to Alberta are Philippines, India, and Nigeria, with corresponding rates of TB ranging from 199 to 638 cases per 100,000 people (**Figure 2**) ((15)). In 2021, 241 people in Alberta had TBD, with a corresponding crude rate of 5.4 per 100,000 population ((6)).

**Table 1** shows the top countries of origin of new arrivals to Alberta from 2016–2021. **Table 2** shows the top five countries of origin and five-year average TB incidence in those countries for PBOC diagnosed with TB in Alberta from 2016–2021 during which time a total of 1,248 people were diagnosed with TB.

**Table 1:** Top 5 countries of origin of new arrivals in Alberta, 2016–2021

Country	n (%)
Philippines	47,605 (24.6)
India	31,810 (16.5)
Nigeria	9,840 (5.1)
China	9,495 (4.9)
Syria	7,300 (3.8)
Total in Alberta	193,130 (100.0)

**Table 2:** Top 5 countries of origin for people born outside the country diagnosed with tuberculosis in Alberta, 2016–2021

Country	n (%)	Average five year incidence
Philippines	414 (33.2)	577/100,000
India	255 (20.4)	201/100,000
Ethiopia	79 (6.9)	133/100,000
Somalia	74 (5.3)	254/100,000
Vietnam	31 (2.5)	174/100,000

## Methods

The enhanced TBI screening initiative for migrants focuses on TBI-test-positive individuals at high risk of reactivation who would benefit from TB preventive therapy (TPT). Individuals coming from select countries are required to complete an immigration medical exam (IME) to evaluate their health in order to be admissible to Canada ((16)). All permanent residents and some temporary resident applicants are required to complete an IME. After screening, applicants who can be part of this intervention must have a complete IME with at least one high-risk medical condition for TB reactivation (HIV/AIDS and have a solid organ transplant, end-stage renal disease, recent close TB contact within five years, and head and neck cancer) together with either a positive interferon-gamma release assay (IGRA) or Tuberculin Skin Test (TST) ((17)). The IRCC notifies provincial or territorial public health authorities of these individuals so that they can arrange assessment for TPT. A letter is also provided at the port of entry to applicants whose IME was performed overseas directing them to follow up with TB services in their intended province/territory of residence within 30 days. As a result, health system contact can be initiated either by provincial/territorial TB services or by the individual. Assessment by a clinician or public health designated specialist is a condition of entry that can affect future eligibility of Canadian citizenship ((12)). For this reason, TB services and individual migrants assume mutual responsibility for medical surveillance of TB in Canada, and compliance is high.

Other high-income countries with low rates of TB in the general population, such as the United Kingdom ((18)), Australia ((19,20)), and the United States ((21)), run varied TB screening programs for inbound migrants to reduce imported prevalent TB infection and disease, as shown in **Table 3**. Compared to Canada, the governments of the United Kingdom, Australia, and especially the United States have more comprehensive TB screening programs for PBOC that emphasize prevention and involve more robust testing of persons arriving from high incidence countries. The initiative evaluated in this article is intended to lessen existing TB prevention gaps between Canada and other high-income countries.

**Table 3:** Basic characteristics of screening programs for migrants in Canada, the United Kingdom, Australia and the United States^a^

Characteristics	Canada	United Kingdom	Australia	United States
Target population	Individuals applying for permanent resident status and select temporary residents who require an IME ((7)).	Visa applicants aged ≥11 years, coming from countries with a TB rate of >40 per 100,000 and staying for ≥6 months ((18)).	Individuals applying for permanent resident status and select non-permanent residents who require an IME ((19,20)).	Immigrants, refugees, or other legal permanent residents ((21)).
Screening tests	Tests include physical examination, medical history, chest X-ray, and sputum for acid-fast bacilli smear and culture if indicated ((7)).	Tests include chest X-ray and symptom inquiry. Sputum for acid-fast bacilli smear and culture is required for those with a suggestive chest radiograph.	Tests include chest X-ray and symptom inquiry. Children aged >2 but <11 years coming from high-TB-incidence countries, which includes all those not categorized by WHO as low-TB risk, are required to complete an IGRA or TST.	Tests include physical examination, medical history, chest X-ray and sputum for acid-fast bacilli smear and culture if indicated. IGRA test for those aged ≥2–15 years, expanding in fall of 2024 to those aged >15 years, who come from high-TB-burden countries (defined by an incidence of ≥20 cases per 100,000).
Medical surveillance requirement	Past history of TB or abnormal chest X-ray, but no microbiological confirmation of disease which are required to report to a public health authority within 30 days of arrival.	Migrants arriving by unofficial routes are screened for active TB at the first point of contact with healthcare services.	Past history of TB or an abnormal chest X-ray but not microbiological confirmation of disease, which are required to report to public health within 28 days of arrival.	Panel physicians assign applicants into one of seven TB classifications with varying travel clearances: Those classified as A or B are referred to their local state health department for follow-up within 90 days of arrival.
Other screening	Selective TBI testing predates the 2019 enhanced program, and applies to individuals who intend to work, study or train in certain areas, including medicine and allied health (16).	TBI testing and treatment for recently arrived (within 5 years) PBOC aged 16–35 from countries with an incidence rate of 150 per 100,000 or greater ((18)).	Selective TBI testing of individuals aged ≥15 years, arriving from high TB-incidence countries who intend to work, study, or train in health care, aged care or disability care.	Refugees undergo domestic screening within 90 days of arrival to find TB disease that may have developed between an overseas IME and arrival to the United States.

Abbreviations: IGRA, interferon-gamma release assay; IME, immigration medical exam; TB, tuberculosis; TBI, tuberculosis infection; TST, tuberculin skin test; WHO, World Health Organization

^a^ Individuals diagnosed with active tuberculosis disease during their immigration medical exam are required to submit proof of treatment completion before being permitted to enter any of the four countries or else have visa conditions lifted

### Implementation and effectiveness

Between 2019 and 2023, IRCC made 9,887 referrals to TB services in Alberta and between May 2019 and May 2023, with 179 or fewer than 2%, resulting from the enhanced TBI screening initiative. To describe the effectiveness of this initiative (i.e., its ability to connect eligible individuals to TB services for domestically delivered prevention), this study focused on TB outcomes. Data were extracted in a retrospective review of public health records to establish care cascades. Every referred individual had a one-year follow up to assess for development of TBD. The characteristics of individuals referred through this initiative are described in this article. Thereafter, to identify implementation challenges, the entire process is explored in detail, from application of screening inclusion to referral and subsequent stages of TB care in Alberta.

## Results

Alberta TB services received 179 referrals through IRCC’s enhanced screening initiative, relating to 177 unique individuals over four years. Characteristics of individuals referred are shown in **Table 4**. The majority of IMEs were performed overseas compared to Canada (71.8% and 28.2%, respectively). Recent close contact of a TB case was the top reason for referral, followed by HIV/AIDS (46.9% and 35.6%, respectively). The majority of referred individuals were applying for permanent residence status (67.2%). Approximately 40% of the individuals referred through the program were coming from the Philippines. Overall, 87.6% of referred individuals came from the World Health Organization (WHO) designated, high-TB-burden countries ((22)). The IGRA was used to screen the majority of individuals, compared to TST (81.4% and 15.2%, respectively). Close to one-quarter of the results of the screening test were reported in the IME qualitatively.

**Table 4:** Characteristics of the 177 unique individuals referred by Immigration, Refugees and Citizenship Canada to Alberta tuberculosis services, May 2019–May 2023^a^

Characteristics	n (%)
**Location of IME**	
Overseas	127 (71.8)
Canada	50 (28.2)
**Screening inclusion group**	
Recent close TB contact (within five years)	83 (46.9)
HIV/AIDS	63 (35.6)
End-stage renal disease	22 (12.4)
Previous head/neck cancer	6 (3.4)
Previous organ/transplant recipient	3 (1.7)
**IME screening tool**	
IGRA	144 (81.4)
TST	27 (15.2)
No test	6 (3.4)
**Reporting of results in IME (n=171)**	
Quantitatively	128 (74.9)
Qualitatively	43 (25.1)
**Gender**	
Male	90 (50.8)
Female	87 (49.2)
**Age group (years)**	
5–14	12 (6.8)
15–35	73 (41.2)
36–60	63 (35.6)
60+	29 (16.4)
**Immigration type^b^**	
Permanent resident	119 (67.2)
Temporary resident	57 (32.2)
**Country of birth**	
Philippines	71 (40.1)
Ethiopia	15 (8.5)
India	15 (8.5)
Nigeria	11 (6.2)
Other	65 (36.7)
**WHO region**	
Western Pacific	78 (44.1)
African	59 (33.3)
South-Eastern Asia	16 (9.0)
Eastern Mediterranean	15 (8.5)
Region of the Americas	8 (4.5)
European	1 (0.6)
**WHO high-burden country^c^**	
Yes	155 (87.6)
No	22 (12.4)

Abbreviations: IGRA, interferon-gamma release assay; IME, immigration medical exam; TB, tuberculosis; TST, tuberculin skin test; WHO, World Health Organization

^a^ Two individuals were referred twice through this initiative

^b^ Immigration type of one individual was unknown

^c^ List of 30 countries created by WHO, which is valid until 2025 ((22))

No one referred through the program and offered treatment developed active TB, whether they accepted and completed treatment, declined, or discontinued, but one individual had prevalent active TBD diagnosed at their surveillance appointment, which occurred within two weeks of landing in Canada.

**Figure 3:**
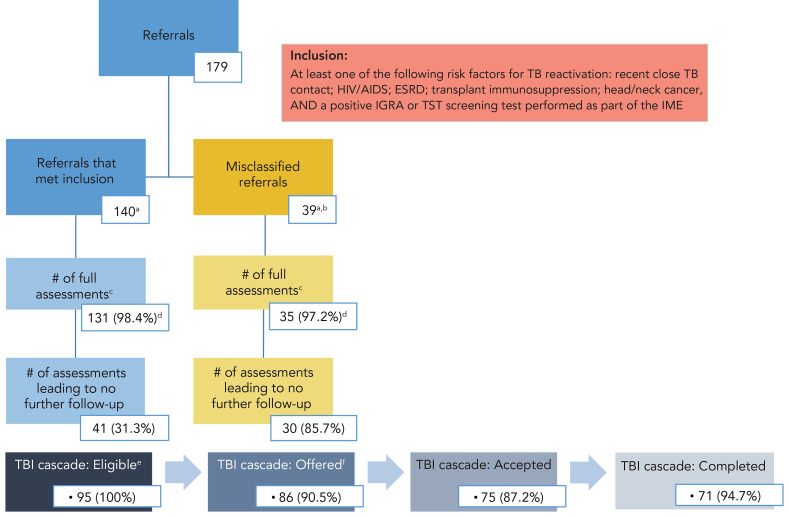
Flow chart illustrating how the 179 referrals moved through the various stages of care in the Alberta tuberculosis program and resulting tuberculosis infection cascades of care for individuals^a,b,c,d,e,f^ Abbreviations: ESRD, end-stage renal disease; IGRA, interferon-gamma release assay; IME, immigration medical exam; TB, tuberculosis; TBI, tuberculosis infection; TST, tuberculin skin test ^a^ Each referral group includes one individual who had two distinct IMEs during the review period and was hence referred twice ^b^ 33 negative test results on file; 6 missing test results ^c^ Of the 166 full assessment appointments, 61 were for individuals who had contact with provincial TB services that pre-dated the referral, of which 72.1% resulted from an IME performed in Canada and 27.9% were from those whose IME was done overseas ^d^ Of surveillance appointments (n=133 in the “met inclusion” group; n=36 in the misclassified group) ^e^ Sum of individuals who were fully assessed but not put to no further follow up ^f^ Nine individuals were not offered treatment for miscellaneous reasons (did not have insurance, died incidentally, moved or left Canada)

Attendance at the surveillance and assessment appointments was high, at 94.3% and 92.7%, respectively (defined in **Table 5**). The median time between surveillance and assessment appointments was 28 days (interquartile range [IQR]: 9, 101), and 10% of all referrals were associated with a wait time of >6 months between referral and assessment. From 177 unique individuals referred, only 95 (53.6%) were ultimately determined to be candidates for prevention, 86 (90.5%) of whom were offered treatment. Just over 87% of individuals who were offered accepted treatment and completion was high at 94.6% (**Figure 3**). Out of 95 individuals referred through this initiative, 71 successfully progressed through the TBI cascade of care while attrition at some step or another affected 24. Individuals who had a successful cascade were slightly younger than those who did not (average 36 years vs. 52.8 years), and less likely to have had their IME in-country (32.4% vs. 58.3%); data not shown.

**Table 5:** Metrics of performance for the enhanced screening initiative showing the cascade of care and redundancies

Performance metrics	n (%)
**Total unique individuals referred**	**177 (100.0)**
Individuals who attended a surveillance appointment^a^	167 (94.3)
Individuals who completed an assessment appointment^b^	164 (92.7)
Individuals who had TBI testing repeated	113 (63.8)
**Total unique individuals who attended surveillance**	**167 (100.0)**
Individuals with a surveillance appointment that preceded referral	60 (35.9)
Individuals whose surveillance appointment occurred within 6 months of referral	89 (53.2)
Individuals whose surveillance appointment occurred >6 months from referral	18 (10.7)
**Total unique individuals who completed physician assessment**	**164 (100.0)**
Individuals recommended TPT	86 (52.4)
Individuals who initiated TPT	75 (87.2)
Individuals who completed TPT	71 (94.7)

Abbreviations: TBI, tuberculosis infection; TPT, tuberculosis preventive therapy

^a^ Surveillance appointment is the first point of contact between the referred individual and the TB program, where an individual may be required to undergo additional testing for TBI

^b^ At an assessment appointment, which follows surveillance, a physician goes through the test results to determine whether an individual would benefit from treatment

Notably, 60 (35.9%) individuals had contact with TB services prior to notification to the province by IRCC of their referral through this initiative, with the median number of days between those events being 148 days (IQR: 67, 364) (see Table 5). For 53 (88.3%) of those individuals, their prior contact occurred more than 30 days before the province receiving their referral, and majority was observed in individuals whose IME was performed in-country (83%). In addition, TBI screening tests were repeated in Alberta for 113 (63.1%) of all individuals referred. Among those, 39 had a negative test result or else no evidence of a prior test having been performed as part of the IME; see Figure 3. From the remaining 140 referrals, after excluding those 39 with negative or no test result in their IME, were two individuals each referred twice. In other words, of the 179 total referrals made, 138 unique individuals were seen from referrals to Alberta TB services that met all inclusion criteria of this enhanced screening initiative over the review period.

## Discussion

This enhanced TBI screening initiative has been nationally administered by IRCC since 2019 but, given its restrictive screening inclusion, applies to very few of all newcomers to Canada ((17,23)). Outcomes of individuals referred through it have heretofore not been reported, a knowledge gap that this evaluation contributes to closing. Local data showed that this initiative was highly effective at connecting referred individuals to TB services in the province in a timely fashion, including for one individual who had prevalent disease at their surveillance appointment who was rapidly provided TBD treatment. Despite this success, only about half of those referred for prevention were considered suitable candidates for TPT after in-country assessment. Those who were offered treatment had high rates of acceptance and completion. The conclusion drawn is that the program was limited by certain inefficiencies, but future evaluations should be undertaken in other high immigrant receiving provinces to determine unique and common obstacles to its implementation and effectiveness with respect to TB control in Canada. From the limited vantage point of this study, cautious, but generalizable takeaways are presented.

First, redundant patient referrals were observed. About one-third of individuals referred by this initiative had already been seen by TB prevention and care services in Alberta prior to the referral being made. This may be due to an administrative delay, whereby individuals initiate their surveillance appointment prior to TB services being notified of the referral. It may also result from in-country applicants, who are likelier to have prior health system contact to manage their high-risk medical condition that satisfies the requirement for screening during the IME. Across Canada, it is the standard of care to screen for TBI among patients provided care for HIV/AIDS, end-stage renal disease, solid organ transplant, and head and neck cancers and this may account for much of the duplication ((9,24)).

Second, a substantial number of individuals referred, who did not meet the screening or test result inclusion criteria, were observed. Such misclassification occurs in the events prior to referrals being made by IRCC, so its rate is likely countrywide. As a result, a high volume of individuals who are not suitable candidates for TPT are referred nationally contributing to increased workload, unnecessary testing, and reduced yield of the intervention.

Third, information management pitfalls and resource waste were observed. For example, in Alberta, nearly two–thirds of individuals referred underwent local repeat screening. On the one hand, this may be due to test results being hard to find. On the other hand, it may be due to misalignment between panel physician member guidance and the local standard of care. For instance, the instructions guide physicians to report test results qualitatively, which conflicts with the standard of care in Alberta to base a TBI diagnosis on quantitative test results ((17)).

Although it is recognized that this initiative is a step in the right direction to close prevention gaps for migrants to Canada, more expansive screening for PBOC especially designed to reach migrants not identified by current methods should be considered. For example, the latest edition (8^th^) of the Canadian Tuberculosis Standards recommends TB screening within five years of arrival for PBOC originating from countries with a TB incidence of >200/100,000, who have low to moderate risk of TB reactivation, and are aged ≤65 years; the TBI screening infrastructure now in place for the IME would ideally support implementation of this recommendation. This strategy would elevate weighting of exposure and infection risk in addition to, or instead of, underlying reactivation risks ((7)). Relatedly, it was noted that individuals whose IME was performed in-country as opposed to overseas were less likely to be considered eligible for prevention at assessment, and more likely to have an inferior TBI care cascade. As a result, cost savings may be achieved by restricting TBI screening to those undertaking an IME overseas.

## Limitations

Although the enhanced TBI screening initiative for migrants is a nationally administered initiative, it was only evaluated in one province and the review period overlapped with the COVID-19 pandemic, which contributed to a sharp decline in global movement and thus reduced expected referral volume. It is noted that while some implementation challenges are related to pre-referral events in administering and reporting of tests in the IME, others may be unique to the organization of TB services by jurisdiction, thereby limiting the generalizability of our reported data. Alberta TB services are highly centralized, with one point of contact for IRCC referrals that get distributed to its three public health TB clinics based on the referred individual’s residence ((25)). Other areas with decentralized TB control efforts may see distinct and more diffuse challenges. The retrospective nature of data collection for this study, and a lack of qualitative data, limit this article to a description of that but not why events occurred. Nevertheless, an evaluation of this initiative is important for detailing implementation lessons that can be used to optimize both its administration, nationally, and corollary patient care, provincially/territorially.

## Conclusion

In low TB-incidence settings like Canada, reactivation of imported infection is a significant driver of the epidemic. Immigration pathways are good places to implement screening as they reflect a major pipeline through which infection flows into Canada. That said, targeting screening, so as not to overwhelm the resources of local TB programs to deliver a treatment response, is crucial; IRCC has implemented one such targeted effort cross-country ((10,26,27)). Compared to its peers, TBI screening has been less robust in Canada. This new initiative, however, is a good step toward expanding TBI screening among PBOC, but we note areas where its administration and local prevention responses can be improved. A lot of work remains if Canada is serious about meeting its TB elimination targets.
